# Which patients with lower respiratory tract infections need inpatient treatment? Perceptions of physicians, nurses, patients and relatives

**DOI:** 10.1186/1471-2466-10-12

**Published:** 2010-03-11

**Authors:** Claudia Baehni, Sabine Meier, Pamela Spreiter, Ursula Schild, Katharina Regez, Rita Bossart, Robert Thomann, Claudine Falconnier, Mirjam Christ-Crain, Sabina De Geest, Beat Müller, Philipp Schuetz, the ProHOSP study group

**Affiliations:** 1 Department of Internal Medicine, University Hospital Basel, Basel, Switzerland; 2 Department of Internal Medicine, Kantonsspital Aarau, Aarau, Switzerland; 3 Institute of Nursing Science, University of Basel, Basel, Switzerland

## Abstract

**Background:**

Despite recommendations for outpatient management, low risk patients with lower respiratory tract infections (LRTIs) are often hospitalized. This survey analyzed perceptions of physicians, nurses, patients and relatives about feasibility of outpatient management and required duration of hospital stay.

**Methods:**

We performed a prospective, observational questionnaire survey in hospitalized patients with LRTI as part of a multicenter trial. Treating physicians and nurses, patients and their relatives were asked on admission and before discharge about feasibility of outpatient treatment over 5 dimensions (medical, nursing, organizational factors, and patients' and relatives' preferences) using continuous scales.

**Results:**

On admission, 12.6% of physicians, 15.1% of nurses, 18.0% of patients and 5.2% of relatives believed that outpatient treatment would be possible. Before hospital discharge, 31.1% of physicians, 32.2% of nurses, 11.6% of patients and 4.1% of relatives thought that earlier discharge would have been feasible. Medical factors were the most frequently perceived motives for inpatient management. These perceptions were similar in all LRTI subgroups and independent of disease severity and associated expected mortality risks as assessed by the Pneumonia Severity Index (PSI).

**Conclusion:**

Independent of type and severity of respiratory tract infection, the misperceived high severity and expected mortality and morbidity were the predominant reasons why treating physicians, nurses, patients and their relatives unanimously believed that inpatient management was necessary. Better assessment and communication about true expected medical risks might contribute to a pathway to shorten in-hospital days and to introduce a more risk-targeted and individually tailored allocation of health-care resources.

**Trial Registration:**

NCT00350987

## Background

Lower respiratory tract infections (LRTIs), including community-acquired pneumonia (CAP), acute bronchitis and exacerbation of chronic obstructive pulmonary disease (ECOPD) are the most prevalent, the most frequently fatal and the most expensive infectious diseases in western countries [1-3]. Inpatient management is approximately 8 to 20 times more expensive than outpatient treatment, but is preferred by many patients and physicians [4-8]. Hence, safely reducing the number of inpatient days and encouraging outpatient treatment improves the quality of care and is cost-effective [7]. Current guidelines recommend to hospitalize patients with an increased risk for adverse medical outcome and to discharge patients as soon as clinical stabilization is documented [9-11]. For this purpose, the Pneumonia Severity Index (PSI) is a well validated prediction tool based on age and medical factors to identify patients at low risk for 30-day mortality and thus suitable for outpatient management[12,13]. However, despite recommendation of this prediction tool by professional organizations, in daily practice low risk patients are often treated as inpatients [8]. Obviously, other factors than evidence-based medical parameters embedded in the PSI - such as nursing and organizational factors, preferences and beliefs of patients and their relatives - strongly influence the rate of hospital admissions and the duration of hospital stays[14,15]. Smaller, previous studies have shown that patients' and relatives' preferences for site of care are important and are influenced by beliefs about safety (fear of rapid deterioration at home or acquiring an infection in hospital), family burden, access to support, or confidence in home-care services [14-16]. Still, the complex interplay of different perceptions towards outpatient management and the impact on site-of-care decisions and length of hospital stay remains largely unknown.

To better understand perceptions of physicians, nurses, patients and relatives about the need for inpatient management of different types and severities of LRTIs, we conducted a prospective survey in patients enrolled in the multi-center ProHOSP trial [17,18] about the perceived necessity for inpatient management and necessary length of hospital stay.

## Methods

### Study Participants

Data for this study were collected from patients enrolled in the multicenter ProHOSP study during the first winter season (between October 2006 until June 2007) who were able and willing to participate[17,18]. Outpatients and patients with severe dementia, language restrictions, immediate need for ICU admission and/or immediate fatal outcome were not included. In addition to patients, we also surveyed the patients' physicians, nurses, and relatives.

A detailed description of the ProHOSP study and baseline characteristics of the participating hospitals has been published previously [17,18]. In brief, patients with clinically suspected LRTIs were consecutively included from October 2006 to March 2008 in six hospitals in Switzerland (range of hospital beds: 252 - 694). LRTI was defined as at least one respiratory symptom (cough, sputum production, dyspnea, tachypnea, pleuritic pain) plus one auscultatory finding or sign of infection (core body temperature >38.0°C, shivers, leukocyte count >10 G/L or <4 G/L cells) independent of antibiotic pre-treatment [9-11,19,20]. CAP was defined as a LRTI along with a new or increasing infiltrate on chest radiograph [9,10]. In the absence of focal chest signs or an infiltrate on chest X-ray, either acute bronchitis [9,10] or exacerbation of COPD was considered. Exacerbation of COPD was defined as a FEV1/FVC ratio below 70% in post-bronchodilator spirometry. Inclusion criteria for patients were age = 18 years with a LRTI <28 days of duration. Patients without informed consent, with severe immunosuppression or chronic infection, intravenous drug users, patients who had been hospitalized within the previous 14 days, as well as patients in a terminal stage of a disease, were excluded.

The primary aim of the ProHOSP study was to compare medical outcomes of patients with LRTIs who were treated with antibiotics according to enforced evidence-based guidelines (control group) with those treated according to a previously tested [21-24] procalcitonin algorithm (intervention group). A predefined secondary outcome was the assessment of medical and non-medical factors that influence hospital admission and length of stay by the use of a systematic survey questionnaire.

The study was approved by all local Ethic Committees and registered in the Current Controlled Trials Database (ISRCTN95100877).

### Study flow

A resident of the Emergency Department (ED) supervised by a board-certified specialist in internal medicine examined patients on admission and randomized patients to receive antibiotics based on a procalcitonin algorithm or based on guideline recommendations using a centralized password-secured website. This website provided all study-related information including patient flow, study algorithms and guidelines for antibiotic therapy based on the latest recommendations [9-11,19,20]. In patients with CAP, a prognostic assessment was conducted on admission and the PSI score was calculated on the basis of the patients' unique set of prognostic indicators as described elsewhere[12]. In both groups, hospitalized patients were clinically reassessed on days 3, 5, 7 and on the day of discharge to evaluate the course of disease. Based on guideline recommendations, discharge was considered if oral intake was feasible, vital signs were stable >24 h, no evidence of acute serious co-morbidity necessitated hospitalization, and the patient achieved pre-admission mobility state[9-11,19,20].

### Measures: Perceptions towards Outpatient Management

To study perceptions towards outpatient management, a standardized questionnaire regarding factors necessitating inhospital treatment based on known factors from the literature [14-16] and personal experience of the nursing and the medical staff was developed through rounds of consensus conferences within the research team. To assure appropriateness of the questions, extensive interviews were performed in a small subset of patients and answers were compared to the survey results. To assess content validity and clarity of the items, the survey was reviewed by independent physicians, nurses, patients and relatives. The feedback was then incorporated into the questionnaire and items were reworded until ambiguous phrasing was sorted out.

In order to obtain estimations about necessity of hospitalization at the time point of patients' admission, and estimations about needed length of stay at the time point of discharge, we performed two interviews: first, a forward-looking questionnaire was completed upon admission focusing on the necessity of hospitalization; second, a similar but backward-looking questionnaire was completed before hospital discharge. The questionnaires were used to collect information from patients, relatives, physicians and the nursing staff. It was designed to document perceptions towards the feasibility of outpatient management over 5 predefined dimensions (medical, nursing, organizational factors, and patients' and relatives' preferences). Thereby, patients' and relatives' preference was defined as the request of patients or relatives with or without specified reasons. The importance of each of these factors had to be quantified on a continuous scale ranging from 0-100% (not important - very important). In addition, these 5 factors were further subdivided into: (a) medical factors (severe infection, expected adverse outcome, relevant comorbidities, need for diagnostic workup, need for intravenous therapy, others); (b) nursing factors (need for support in activities of daily living, with oral drug intake, with personal hygiene, mobility, others); (c) patient specific factors (patients preference); (d) relatives specific factors (no capacity to take care of the patient, relatives preference); and (e) organizational factors (waiting for approval of health insurance coverage, waiting for placement in nursing home, organization of outpatient nursing care ("Spitex"), inhospital organizational reason, preparation of discharge and others). The specific questionnaire items are displayed in Additional file 1.

Data for the current study were collected by trained study nurses, research fellows and medical students. Within 3 days of admission to hospital they interviewed patients, one of their relatives in case they played an active role in the decision making about hospitalization and if it was possible to contact them, the treating physician and the nurse in charge. Within 3 days before hospital discharge the same 4 parties were re-interviewed using the same questionnaire, but from a backward-looking perspective. At that time-point, it was requested to estimate how many days the hospital stay could have been shortened.

### Other Variables

The PSI score categorizes patients into 5 classes: those in class I-III (PSI = 90) designate lower risk patients with an estimated mortality of <1-3% and those in class IV-V (PSI > 90) represent high risk individuals with an estimated mortality of around 10% and 30%, respectively[12]. As outlined by guidelines, the PSI is an effective tool to support physicians in taking decisions regarding patient management [9-11].

### Statistical Analysis

In a first step, we calculated descriptive statistics of baseline characteristics within 4 patients' groups according to their underlying diagnoses (low risk CAP (PSI I-III), high risk CAP (PSI IV-V), ECOPD and acute bronchitis); thereby, results are presented as frequencies (%) or medians (interquartile range, IQR) and multigroup comparisons were done with Kruskal-Wallis or Chi-Square tests as appropriate. In a second step, we analyzed the results of the questionnaire within the 4 diagnoses groups and present data as frequencies (%) and graphically display them. Multigroup comparison of binary data was done with Chi-Square tests.

All testing was two-tailed and p values less than 0.05 were considered to indicate statistical significance. All statistical analyses were performed using STATA 9.2 (Stata Corp, College Station, Tex).

## Results

### Baseline characteristics

This study includes 566 inpatients with LRTIs out of a total of 729 patients enrolled in the first winter season of the ProHOSP study [17,18]. The remaining 163 (22.4%) patients were not included because of outpatient therapy (n = 84), immediate adverse outcome (n = 25) or because they were not able or willing to participate (n = 54). In the participating 566 patients, CAP was diagnosed in 396 (70%), ECOPD in 103 (18%) and acute bronchitis in 45 (8%). In patients with CAP, severity assessment according to the PSI classified 181 as low risk CAP patients (PSI I-III) and 215 as high risk CAP patients (PSI IV-V). The 22 (4%) patients with other final diagnoses than LRTI were not further considered for this analysis, thus the total number of patients analyzed is 544.

The median age of the overall study population was 74 years (IQR 61-82) and 43.9% were females. For the purpose of this study, patients were divided into 4 subgroup categories according to their underlying diagnosis: low risk CAP (PSI I-III), high risk CAP (PSI IV-V), ECOPD and acute bronchitis. Detailed baseline characteristics of the study population including demographic and clinical data, co-existing illnesses and 30-day-outcomes are summarized in Table 1. As expected, high risk CAP patients were older, more often nursing home residents and had higher rates of comorbidities and adverse outcome. The overall 30-day mortality rate was 3.3% (18 out of 544). Mortality was highest for patients with high risk CAP (5.6%), lower for patients with acute bronchitis (2.2%) or ECOPD (3.3%) and lowest for low risk CAP patients (0.6%). The overall length of hospital stay was 9 (IQR 5-13) days; it was highest in high risk CAP (10 (IQR 7-15) days) and ECOPD patients (8 (IQR 5-12) days) and lowest in low risk CAP (7 (IQR 5-11) days) and acute bronchitis patients (6 (IQR 4-10) days) (Kruskal-Wallis P < 0.001). In some patients with immediate adverse outcomes and severest medical condition the completion of the questionnaire was not possible, thus the rate of adverse outcomes is lower as compared to original study population [17]. Randomization of patients had no influence on the length of hospital stay and on the time to adverse outcomes including mortality and was thus not further considered (hazard ratio 0.99 (95%CI 0.84-1.18) and 1.20 (95%CI 0.46-3.11), respectively).

**Table 1:** Baseline data for all patients *(n = 544) *with respect to the underlying diagnosis

					P value**
	**CAP** **Low Risk** ***(n = 181)***	**CAP** **high Risk** ***(n = 215)***	**Acute bronchitis** ***(n = 45)***	**Exacerbation of COPD** ***(n = 103)***	

**Demographics**					
- Age (years, ǂ)*	61 (47-74)	80 (73-85)	73 (60-82)	76 (65-82)	*<0.001*
- Gender (%, male)*	87 (48.1)	138 (64.2)	20 (44.4)	60 (58.3)	*0.004*
- Nursing home resident (%)*	5 (2.8)	16 (7.4)	2 (4.4)	7 (6.8)	*0.14*

**Clinical signs**					
- Systolic Blood pressure (mmHg)*	130 (118-150)	130 (117-146)	136 (120-150)	139 (120-150)	*0.34*
- Pulse rate (bpm)*	98 (80-108)	95 (80-110)	84 (71-100)	91 (82-105)	*0.034*
- Respiratory rate (bpm)*	20 (16-24)	20 (16-30)	20 (16-24)	24 (18-28)	*<0.001*
- Temperature (°C)*	38.2 (37.4-38.8)	38.1 (37.4-38.9)	37.1 (36.7-38.2)	37.2 (36.5-38.0)	*<0.001*

**Comorbidity**					
- Chronic heart failure*	6 (3.3)	64 (29.8)	7 (15.6)	16 (15.5)	*<0.001*
- Cerebrovascular disease*	6 (3.3)	27 (12.6)	4 (8.9)	8 (7.8)	*0.01*
- Renal dysfunction*	15 (8.3)	70 (32.6)	16 (35.6)	19 (18.4)	*<0.001*
- Neoplastic disease*	4 (2.2)	53 (24.7)	6 (13.3)	9 (8.7)	*<0.001*
- Pneumopathy	47 (26.0)	81 (37.7)	0 (0)	103 (100)	*<0.001*
- Diabetes mellitus	22 (12.2)	46 (21.4)	8 (17.8)	21 (20.4)	*0.092*

**30 days outcome**					
- Length of hospital stay (ǂ)	7 (5-11)	10 (7-15)	6 (4-10)	8 (5-12)	*<0.001*
- Mortality (%)	1 (0.6)	12 (5.6)	1 (2.2)	4 (3.9)	*0.051*
- ICU admission (%)	10 (5.5)	25 (11.6)	2 (4.4)	6 (5.8)	*0.063*

*criteria included in the PSI, ǂ median (Interquartile range IQR)

CAP denotes community acquired pneumonia, low risk PSI includes PSI classes I-III, high risk PSI includes PSI classes IV-V, COPD chronic obstructive pulmonary disease, ICU Intensive Care Unit

**P values are calculated from Kruskal-Wallis or Chi-Square tests

### Results of the questionnaire

A total of 180 residents and 62 senior residents cared for the patients at the 6 sites participating. The return rate of the questionnaires on admission and before discharge was 92.1% (n = 501) and 84.5% (n = 460) for physicians, 84.0% (n = 457) and 76.5% (n = 416) for nurses, 84.7% (n = 461) and 77.4% (n = 421) for patients and 27.8% (n = 151) and 26.8% (n = 146) for relatives. Questionnaires were only distributed to relatives if patients agreed, if relatives played an active role in the decision making about hospitalization and if it was possible to contact them.

**Figure 1:**
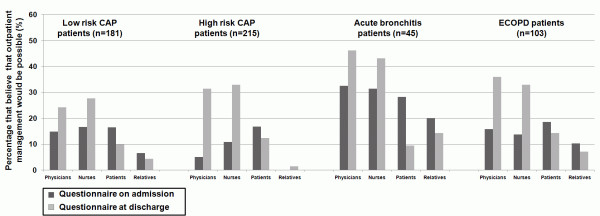
**Estimation if outpatient treatment would be possible on admission (dark grey) and at discharge (light grey)**.

Overall on admission, 12.6% (n = 63) of the physicians, 15.1% (n = 69) of the nurses, 18.0% (n = 83) of patients and 5.2% (n = 8) of the relatives stated, that outpatient treatment would be possible. At hospital discharge, 31.1% (n = 144) and 32.2% (n = 134) of the physicians and nurses declared that earlier discharge would have been possible with a median of 2 (IQR 1-3) and 2 (IQR 2-3) days. In comparison, only 11.6% (n = 49) and 4.1% (n = 6) of patients and relatives declared at hospital discharge that earlier discharge would have been possible with a median of 2 (IQR 1-3) and 2 (IQR 1-2) (Kruskal-Wallis, p < 0.001). Figure 1 shows detailed estimations of physicians', nurses', patients' and relatives' perceptions about possible outpatient treatment on admission and at discharge in subgroups of low and high risk CAP, acute bronchitis and ECOPD patients. In all LRTI subgroups, physicians, nurses, patients and relatives had similar perceptions and stated on admission that in about 10-20% and at discharge in about 20-40% outpatient management would have been possible. Additional file 2 and 3 show more detailed results of the survey in all LRTI subgroups.

**Figure 2:**
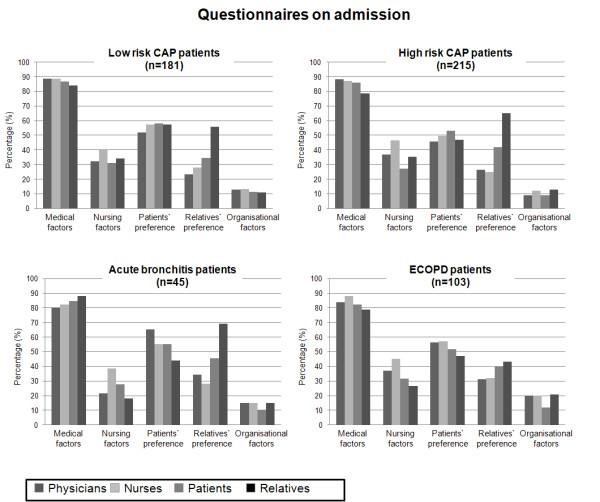
**Reasons for inpatient treatment on admission on a scale from 0-100% in all subgroups of patients**. First column (dark grey) representing physicians, second column (very-light grey) representing nurses, third column (light grey) representing patients, fourth column (black) representing patients' relatives. Of note, the number of patients (n) corresponds to the total number of included patients.

**Figure 3:**
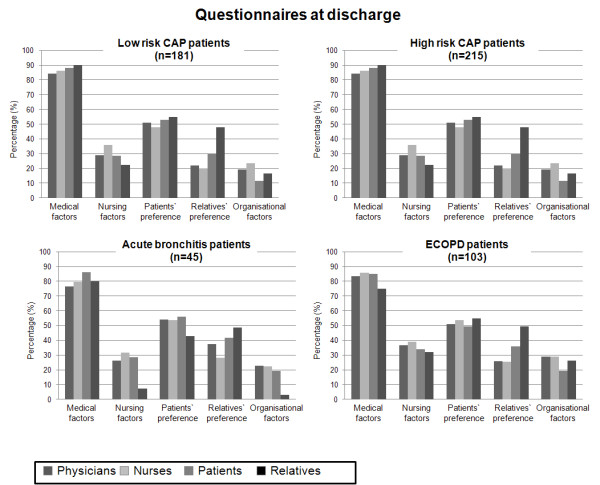
**Reasons for inpatient treatment at discharge on a scale from 0-100% in all subgroups of patients**. First column (dark grey) representing physicians, second column (very-light grey) representing nurses, third column (light grey) representing patients, fourth column (black) representing patients' relatives. Of note, the number of patients (n) corresponds to the total number of included patients.

The principle factors, which made inpatient treatment necessary, had to be quantified on a scale from 0-100% (Figure 2 and 3). Overall on admission and at discharge, and in all LRTI subgroups, medical reasons were perceived to be most important, respectively, by all parties to a similar extent (Kruskal-Wallis on admission p = 0.24; at discharge: 0.32): physicians (86.8% and 81.7%), nurses (87.4% and 83.1%), patients (85.7% and 87.2%) and relatives (80.9% and 85.3%). Following medical reasons, the predominant factors necessitating inpatient management overall and in all LRTI subgroups were patients' preferences, nursing factors, relatives' preferences and organizational factors.

We further asked to specify the main medical factors subdivided into the following categories: fear of severe infection, fear of adverse outcome, severe comorbidities, requiring further diagnostic work up, need for intravenous therapy, other reasons. Overall, fear of severe infection was the most important factor on admission, followed by need for intravenous therapy and comorbidities. Table 2 shows details on medical reasons in all subgroups of patients.

**Table 2:** Main medical factors leading to hospitalization of patients with LRTIs (n = 544)

	Low Risk CAP patients (PSI class I-III)					High risk CAP patients (PSI class IV-V)				
	
	*(n = 181)*					*(n = 215)*				
	**Physicians**	**Nurses**	**Patients**	**Relatives**	***P values***	**Physicians**	**Nurses**	**Patients**	**Relatives**	***P values***
	
	**(n = 167)**	**(n = 154)**	**(n = 158)**	**(n = 45)**		**(n = 200)**	**(n = 182)**	**(n = 178)**	**(n = 67)**	

**Fear of severe infection**	40.1	40.2	53.9	46.7	*<0.001*	35.9	36.3	54.1	50.5	*<0.001*

**Fear of adverse outcome**	5.5	4.6	7.5	8.3	*0.02*	4.7	5.3	4.5	8.4	*0.002*

**Severe comorbidities**	11.3	8.9	11	15	*<0.001*	20.4	14.3	15.7	21.1	*<0.001*

**Requires further diagnostic work up**	8.9	10.8	5.3	1.7	*<0.001*	8.6	9.4	5.4	3.2	*0.03*

**Requires intravenous therapy**	28.8	31.7	15.8	16.7	*0.002*	26.8	29.2	12.4	6.3	*<0.001*

**Other reason**	5.5	3.9	6.6	11.7	*0.24*	3.7	5.6	7.9	10.5	*0.03*

	**Patients with acute bronchitis**					**Patients with ECOPD**				
	
	***(n = 45)***					***(n = 103)***				

	**Physicians**	**Nurses**	**Patients**	**Relatives**	***P values***	**Physicians**	**Nurses**	**Patients**	**Relatives**	***P values***
	
	**(n = 40)**	**(n = 35)**	**(n = 39)**	**(n = 10)**		**(n = 94)**	**(n = 86)**	**(n = 86)**	**(n = 29)**	

**Fear of severe infection**	20	33.3	42.3	38.9	*0.008*	29.7	41.9	51.7	60	*<0.001*

**Fear of adverse outcome**	12.3	13.7	9.6	11.1	*<0.001*	12.7	8.9	9.5	13.3	*0.007*

**Severe comorbidities**	32.3	21.6	25	22.2	*<0.001*	27.2	13.7	20.7	16.7	*<0.001*

**Requires further diagnostic work up**	18.5	7.8	5.8	0	*<0.002*	8.9	8.1	5.2	3.3	*<0.002*

**Requires intravenous therapy**	9.2	15.7	3.8	11.1	*<0.003*	14.6	21.8	6	3.3	*<0.003*

**Other reason**	7.7	7.8	13.5	16.7	*0.001*	7	5.6	6.9	3.3	*0.092*

Similarly, we asked to further specify nursing reasons into the following categories: support in activities of daily living, oral drug intake, personal hygiene, mobility, others. Overall in all subgroups, support in activities of daily living was the most important nursing reason on admission and at discharge necessitating inpatient treatment. Table 3 shows details of nursing factors for all subgroups.

**Table 3:** Main nursing factors leading to hospitalization of patients with LRTIs

	Low Risk CAP patients (PSI class I-III)					High risk CAP patients (PSI class IV-V)				
	
	*(n = 181)*					*(n = 215)*				
	**Physicians**	**Nurses**	**Patients**	**Relatives**	***P values***	**Physicians**	**Nurses**	**Patients**	**Relatives**	***P values***
	
	**(n = 167)**	**(n = 154)**	**(n = 158)**	**(n = 45)**		**(n = 200)**	**(n = 182)**	**(n = 178)**	**(n = 67)**	

**Support in activities of daily living**	54.2	36.8	50.9	36.8	*0.01*	46.9	40.1	43.4	34.2	*<0.001*

**Oral drug intake**	3.4	16.7	12.3	10.5	*0.75*	3.4	9.7	8.5	12.3	*<0.001*

**Personal hygiene**	11.9	18.4	24.6	26.3	*0.7*	18.3	22.8	20.2	21.9	*0.02*

**Mobility**	18.6	13.2	10.5	15.8	*0.08*	22.3	18	20.2	17.8	*0.034*

**Others**	8.5	14	1.8	5.3	*0.16*	2.3	5.6	5.4	8.2	*0.04*

	**Patients with acute bronchitis**					**Patients with ECOPD**				
	
	***(n = 45)***					***(n = 103)***				

	**Physicians**	**Nurses**	**Patients**	**Relatives**	***P values***	**Physicians**	**Nurses**	**Patients**	**Relatives**	***P values***
	
	**(n = 40)**	**(n = 35)**	**(n = 39)**	**(n = 10)**		**(n = 94)**	**(n = 86)**	**(n = 86)**	**(n = 29)**	

**Support in activities of daily living**	45.5	54.2	52.4	33.3	*<0.001*	52.5	34.5	46.7	60	*<0.001*

**Dementia**	3	0	0	0	*0.57*	3.3	1.8	0	6.7	*0.23*

**Oral drug intake**	12.1	12.5	9.5	16.7	*0.01*	4.9	15.9	13.3	13.3	*0.003*

**Personal hygiene**	21.2	12.5	23.8	33.3	*0.03*	14.8	20.4	13.3	6.7	*0.02*

**Mobility**	15.2	8.3	14.3	16.7	*0.03*	19.7	16.8	17.8	13.3	*0.02*

**Others**	3	12.5	0	0	*0.004*	4.9	10.6	8.9	0	*0.23*

## Discussion

Site-of-care decisions in LRTIs are pivotal, yet admission rates and duration of hospital stays vary considerably and appear non-standardized in real-life clinical practice [25,26]. This survey provides new insights into perceptions of physicians, nurses, patients and relatives about possible outpatient management, the perceived necessary length of hospital stay and reasons necessitating inpatient management in patients with different severities of LRTIs. This survey revealed that independently of type of LRTI and expected mortality, most patients with LRTIs are hospitalized because physicians, nurses, patients and relatives all believe that inpatient management is indicated due to medical reasons, particularly fear of severe infection. To a lesser extend nursing reasons and patients' and relatives' personal preferences were mentioned.

It is known that outpatient management has advantages over inpatient management. Patients treated in their home are able to resume normal activities sooner and are less likely to experience complications such as thrombo-embolic events, secondary infections with more virulent and resistant pathogens found in the hospital (nosocomial infections) and intravascular catheter-related infections since antibiotic treatment is usually practiced with an oral antimicrobial agent [27]. In addition, treatment at home is less expensive and studies have demonstrated that low risk CAP patients prefer outpatient to inpatient treatment [8]. Still, as demonstrated in previous studies [4,28,29] as well as in the presented study, many patients with LRTIs are treated as inpatients even if being at a low risk for medical complications. As inpatient management should be considered especially for patients at higher risk for complications and mortality, guidelines recommend that the decision to hospitalize an individual patient with a LRTI should be validated against at least one objective tool of risk assessment[10]. In this regard, the PSI is a valid tool. Patients with a PSI of IV or V are at increased risk for short term mortality, and thus in these patients hospitalization should be strongly considered [10,12,13]. Yet, as demonstrated by others [4,28,29] and confirmed in the present study, a high percentage of low risk patients with PSI I-III are still being hospitalized despite enforced implementation to calculate the PSI and follow its recommendation on admission in our study. Once a patient is hospitalized, guidelines recommend that discharge should be considered if oral intake is feasible, vital signs are stable, if there is no evidence of acute serious co-morbidity requiring hospitalization and if the patient achieves pre-admission mobility[10,11]. Still, substantial regional and national variations of length of stay have to be taken into account, furthermore prolonged hospitalization times depend on disease-related factors, medical complications and non-clinical factors [7]. These observations highlight the importance of physician judgment for the site-of-care decision in patients with LRTIs. In addition, it raises the questions if physician judgment is influenced by strictly medical or, possibly more important other factors such as nursing and organizational factors, preferences and believes of patients and their relatives, and how these factors may interact, respectively.

Interestingly, despite enforced assessment of the PSI during this study, physicians, nurses, patients, and relatives most often justified inpatient management with medical factors, namely fear of severe infection and fear of complications not directly included in the PSI. To a lesser extent nursing factors were also mentioned to be important, mainly support in activities of daily living. Thus, all involved parties intuitively overestimated the disease specific risks. As the PSI is a medical risk assessment tool to be used by physicians, timely information of the nursing staff, patients and relatives about usually lower-than-expected complications would be desirable. In addition, similar tools should be designed for other LRTIs than CAP, namely ECOPD and acute bronchitis. We found a considerable length of hospital stay for all patients and particularly in low risk CAP and acute bronchitis patients. Many of these patients fulfilled discharge criteria from the beginning, but still only 20-40% of physicians and nurses and 10% of patients and relatives believed that earlier discharge would be feasible, mainly because of ongoing fear of severe infection and associated complications. Interestingly, this perception was similar prospectively on admission and retrospectively at discharge. Continuous information about the course of disease, expected risks and the possibility of early discharge may encourage not only physicians, but also nurses, patients and relatives to envisage discharge.

An important finding of this study is that beyond the severity of the infection and expected risks, the need for intravenous antibiotic treatment was often mentioned as a medical reason necessitating inpatient management and justifying a longer length of stay, mainly by physicians and nurses. The regular evaluation of its need and change to oral treatment as soon as possible is thus of outmost importance and could reduce the duration of hospitalization as well.

Interestingly, in this study physicians and nurses were much more confident that earlier discharge would be feasible in about one forth of patients, while patients and relatives were much more reluctant to earlier discharge. In daily practice, patients often don't have enough self-confidence and fear complications of disease. Early information of patients and relatives about convalescence and the diverse possibilities of home nursing and home caring might be an effective strategy to empower patients for a shared decision-making about earlier discharge.

**Figure 4:**
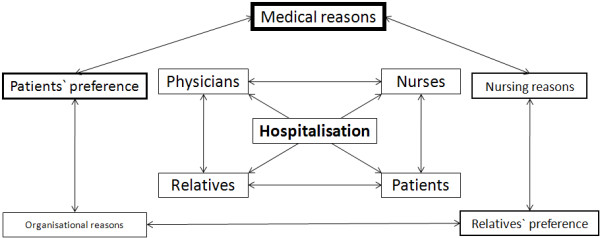
**Different factors which potentially influence the site-of-care decision**.

Consistent with theoretical concepts from Glouberman[14,15], this study shows that not only medical but also non-medical factors influence the site-of-care and discharge decision in patients with LRTIs. Although the initial site-of-care and discharge decision is primarily made by physicians and based on medical reasons, preferences of patients and their relatives, nursing and organizational factors are also considered (Figure 4). Thus, specifically addressing and communicating these "non-medical" factors with everyone involved in patient management might help to optimize caring and curing procedures and to avoid unnecessary hospitalization. Prior studies have demonstrated that outpatient management is often preferred by low risk CAP patients and that most physicians appear not to involve patients in the site-of-care decision [8]. However, in this study, many patients preferred inpatient management due to concerns regarding their medical situation and possible complications. Patients' concerns may be the result of missing information on their low medical risk or miscommunication and do not correspond with findings from large studies[12]. A more explicit education of patients would likely change patients' perspectives and thus indirectly influences the site-of-care decision.

A very recent US study found that low-risk patients with CAP are frequently hospitalized because of comorbid illness, because of requests for hospitalization made by other treating physician, the patient, or the patient's family or the provider's perception that the case of pneumonia was more severe than indicated by the PSI [28]. In our study, comorbid disease was only mentioned in 11% of low risk patients thus not a major driving factor. However, fear of complications and higher perceived risk may prompt patients and relatives to request hospitalization.

To the best of our knowledge, this is the first study to systematically investigate perceptions of physicians, nurses, patients and relatives about possible outpatient management and about medical and non-medical factors that influence this decision. The strengths of this study are the prospective conduct of this survey in a large number of LRTI patients from 6 Swiss Hospitals differing in size and type, including one University hospital and the standardized and enforced risk assessment of patients on admission using a centralized website. Still, some limitations of this study need consideration. Firstly, the survey was not numerically equally conducted in each interviewed group. Questionnaires were only distributed to relatives if they took part in the decision making about hospitalization and therefore relatives are underrepresented. Secondly, older and severely ill patients, as well as some of the relatives may have been overstrained by this survey asking for personal estimations. Similarly, physicians and nurses may not have answered all questions independently and adequately because of time constraints and mutual interaction. Finally, the results of this study may not unconditionally apply to other settings or geographic regions.

## Conclusion

In conclusion, we found that the complication and mortality risks in LRTIs are in general overestimated by physicians, nurses, patients and relatives possibly leading to higher rates of inpatient management and longer hospital stays. We hypothesize that more objective and easy-to-use assessment and information of the medical staff, but especially patients, their relatives and the nursing staff with simpler, more convenient, appropriate and persuasive risk assessment tools and information would be useful. Furthermore, a more explicit discussion between physicians, nursing staff, patients and their relatives about expected risks and existing fears, and the consideration of medical as well as non-medical factors would help to avoid unnecessary admissions, to shorten necessary hospitalizations and to optimize caring and curing procedures, and therefore improve the cost-efficiency of the treatment and optimize the allocation of our limited health-care resources.

## Competing interests

The authors declare that they have no competing interests.

## Authors' contributions

CB, SM, PaS, US, BM and PS had the idea, wrote the protocol and initiated the study. CB, SM, PaS, US, KR, RB, RT, CF, MCC, BM and PS designed the questionnaire, managed the trial and collected data. CB, SM, PaS and PS performed the statistical analyses. CB, SM, PaS, US, KR, RB, BM and PS drafted the manuscript and RT, CF, SDG and MCC amended and commented on the manuscript. All authors approved the final version.

## Pre-publication history

The pre-publication history for this paper can be accessed here:

http://www.biomedcentral.com/1471-2466/10/12/prepub

## Supplementary Material

Additional file 1 **Questionnaire items**. The specific questionnaire items was used within this study to assess perception of physician, nurses, patients and relatives are presented Click here for file

Additional file 2 **Survey results for patients with low and high risk CAP on admission and at discharge**. Detailed results for patients with community-acquired pneumonia (CAP) with low risk PSI classes (I-III) and high risk classes (IV-V); Initial survey (1-3 days after hospitalization) in the upper part, follow up survey (1-3 days before discharge) in the lower part Click here for file

Additional file 3 **Survey results for patients with ECOPD and acute bronchitis on admission and at discharge**. Detailed results for patients with exacerbation of COPD (ECOPD) and acute bronchitis classes (IV-V); Initial survey (1-3 days after hospitalization) in the upper part, follow up survey (1-3 days before discharge) in the lower par Click here for file
