# Supplementary figures and images for: Which patients with lower respiratory tract infections need inpatient treatment? Perceptions of physicians, nurses, patients and relatives
Source: BMC Pulm Med. 2010 Mar 11;10:12. doi: 10.1186/1471-2466-10-12 (PMC2850889; doi:10.1186/1471-2466-10-12)

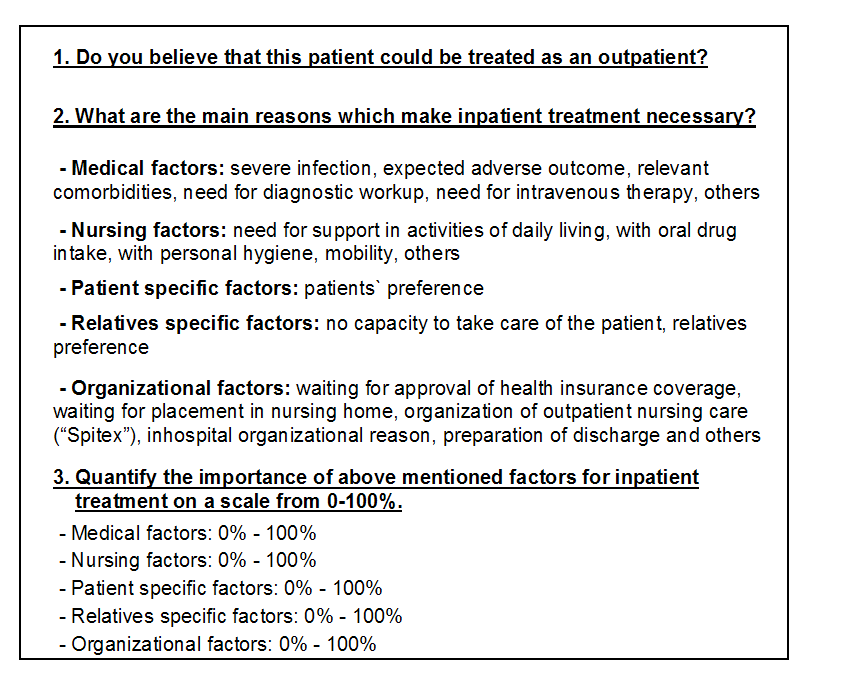

Supplement: Additional file 1 — Questionnaire items. The specific questionnaire items was used within this study to assess perception of physician, nurses, patients and relatives are presented [file 1471-2466-10-12-S1.TIFF]

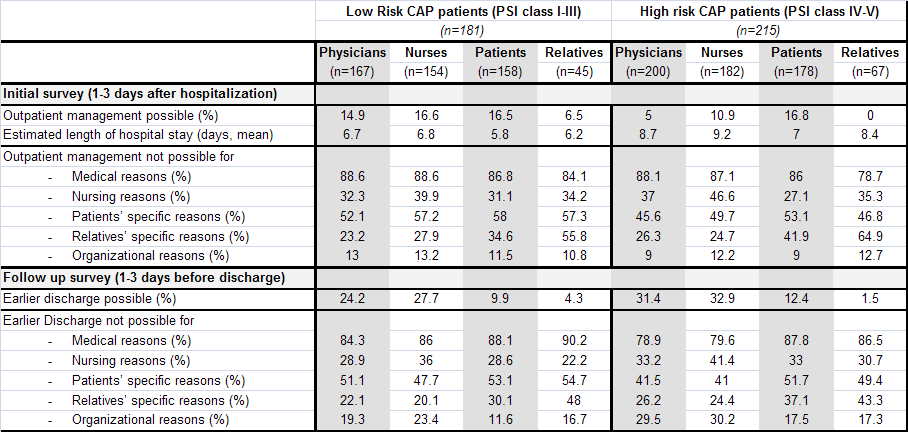

Supplement: Additional file 2 — Survey results for patients with low and high risk CAP on admission and at discharge. Detailed results for patients with community-acquired pneumonia (CAP) with low risk PSI classes (I-III) and high risk classes (IV-V); Initial survey (1-3 days after hospitalization) in the upper part, follow up survey (1-3 days before discharge) in the lower part [file 1471-2466-10-12-S2.TIFF]

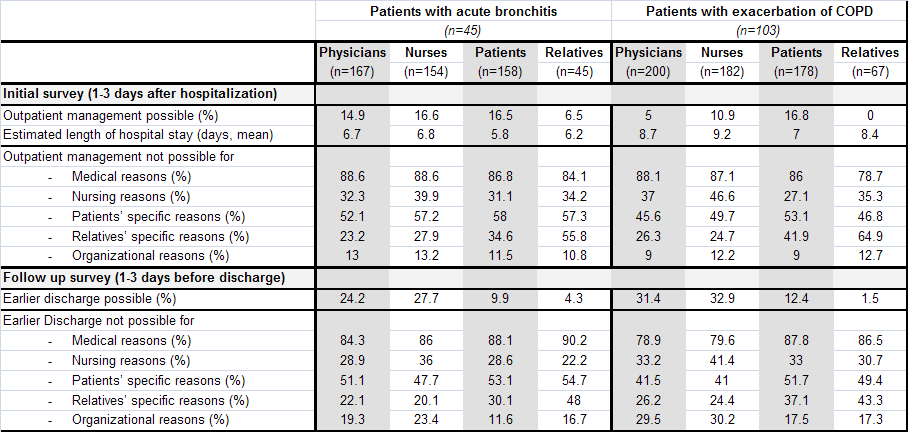

Supplement: Additional file 3 — Survey results for patients with ECOPD and acute bronchitis on admission and at discharge. Detailed results for patients with exacerbation of COPD (ECOPD) and acute bronchitis classes (IV-V); Initial survey (1-3 days after hospitalization) in the upper part, follow up survey (1-3 days before discharge) in the lower par [file 1471-2466-10-12-S3.TIFF]
